# Supplementary material for: Atomic structure of a nudivirus occlusion body protein determined from a 70-year-old crystal sample
Source: Nat Commun. 2023 Jul 13;14:4160. doi: 10.1038/s41467-023-39819-1 (PMC10345106; doi:10.1038/s41467-023-39819-1)
Supplement: Supplementary file 1 — Supplementary Information [file 41467_2023_39819_MOESM1_ESM.pdf]

Supplementary information

Atomic structure of a nudivirus occlusion body protein determined from a 70-year-old crystal sample.

Jeremy R. Keown[1], Adam D. Crawshaw[2], Jose Trincao[2], Loïc Carrique[1], Richard J. Gildea[2], Sam Horrell[2], Anna J. Warren[2], Danny Axford[2], Robin Owen[2], Gwyndaf Evans[2], Annie Bézier[3], Peter Metcalf [4], Jonathan M. Grimes[1]

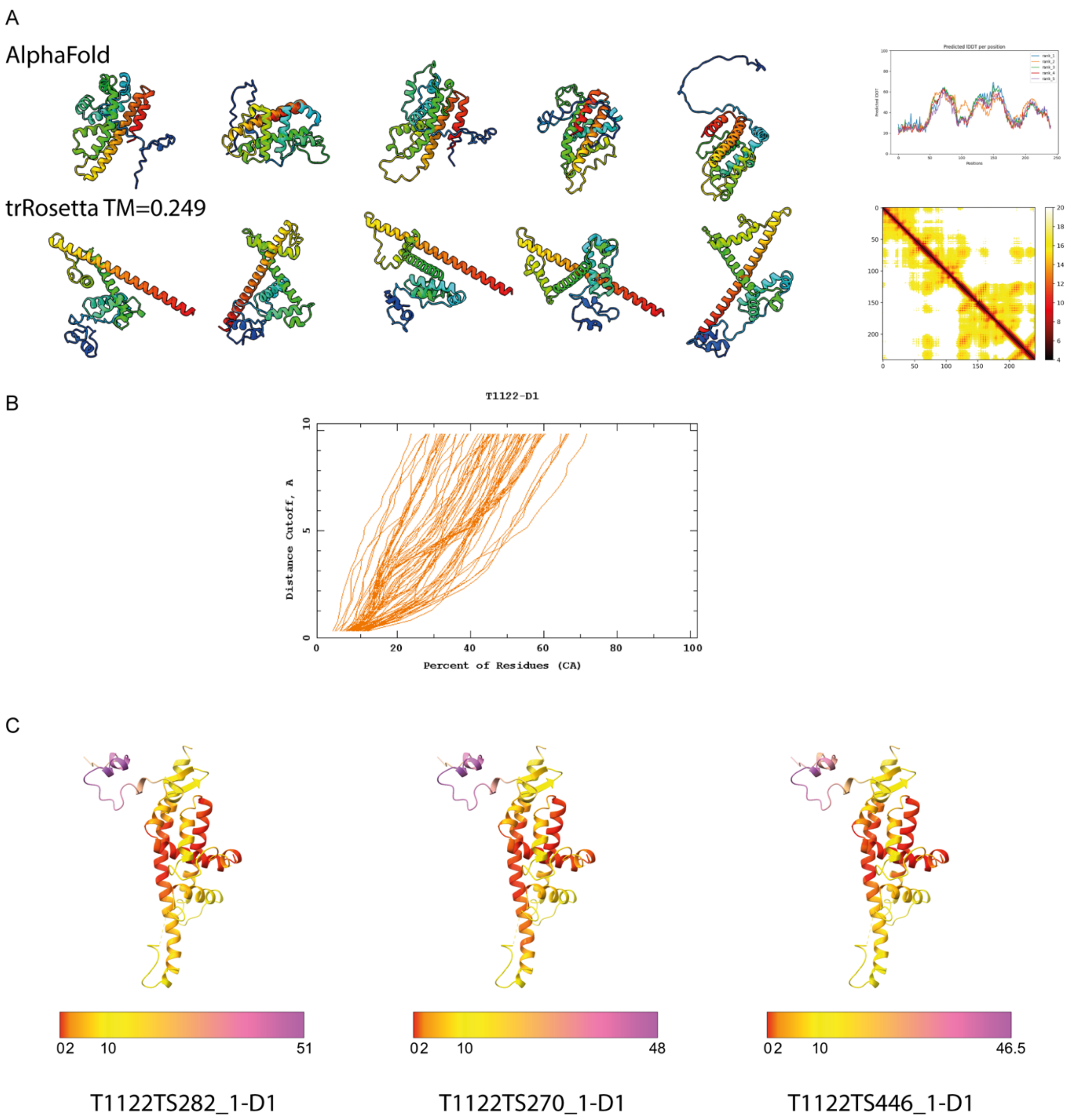

Supplementary Figure 1. **Nudivirus polyhedra predictions.** Structure prediction using Alphafold or trRosetta for (A) ToNV (accession number: YP_009116706.1). Five models were generated with each method and are coloured from the N- to C-terminus (blue-red). Plots and TM showing model quality are included for the prediction. (B) Overview from CASP15 (https://predictioncenter.org/casp15/gdtplot.cgi?target=T1122-D1) showing the percentage of residues within a given distance of their experimental location. (C) Three representative predicted models from CASP15 were aligned to the experimentally determined ToNV polyhedrin structure. The experimentally determined structure is shown and coloured according to the distance between the equivalent Cα in the predicted model (RMSD, units of (Å)).

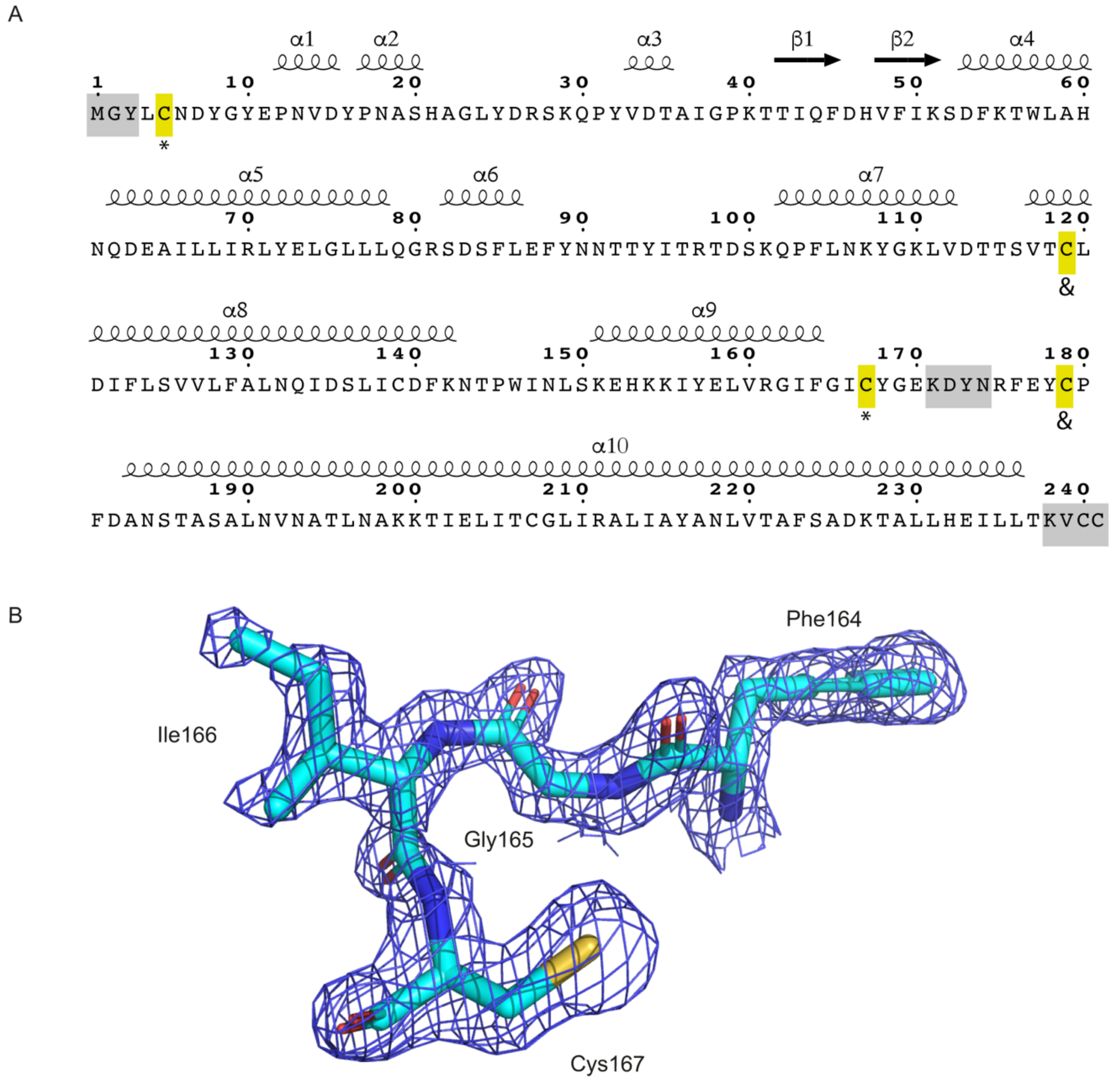

Supplementary Figure 2. **Annotation of secondary structure elements in the native ToNV OB.** ToNV_Orf59 sequence annotated with the secondary structure elements present in the native ToNV polyhedrin (A). Pairs of cysteines are indicated with matching symbols. Residues not resolved in the electron density are indicated in grey. (B) Electron density and model showing the cis peptide between Gly165 and Ile166. Density is shown at $2F_O - F_C$ map at 1σ (blue).

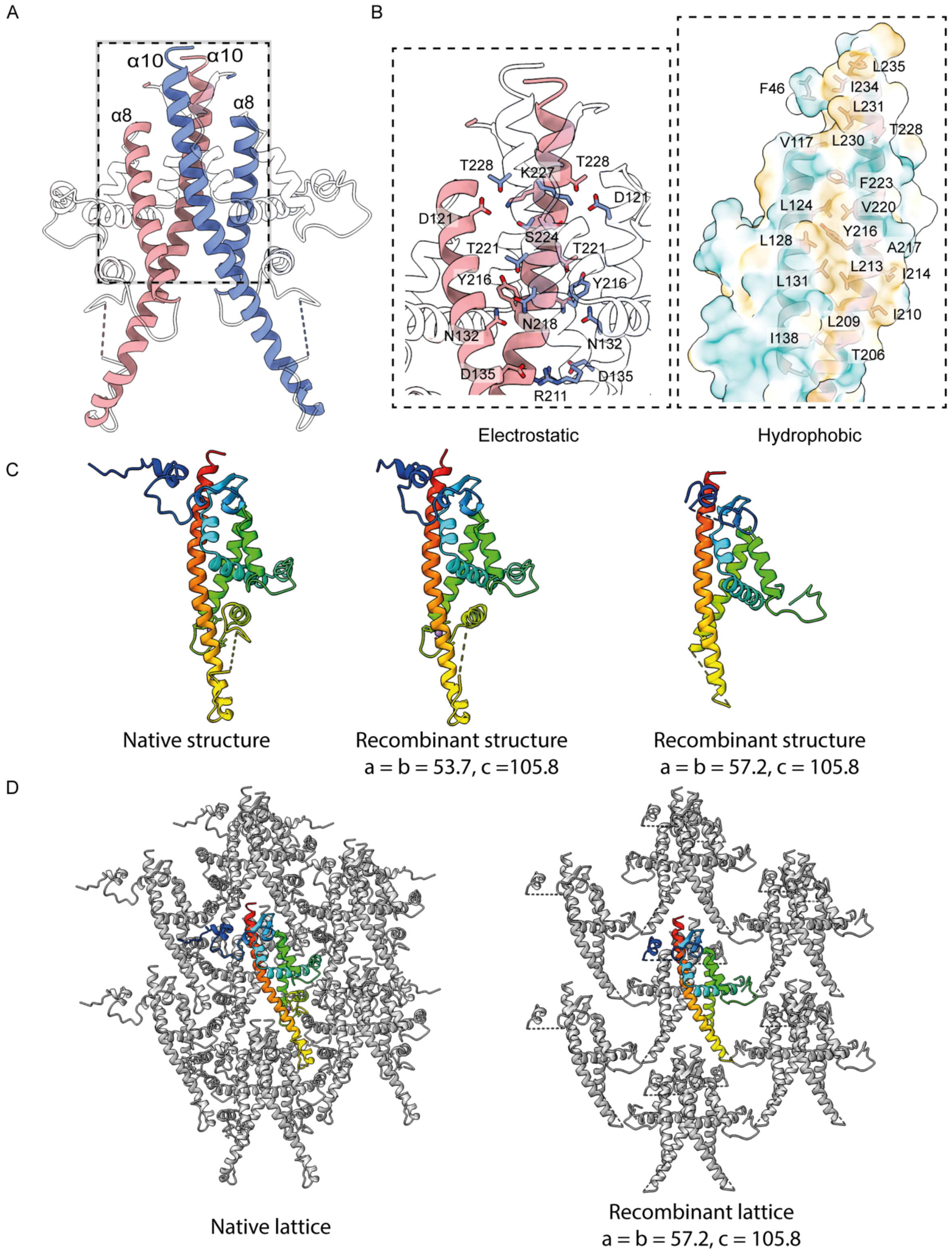

Supplementary Figure 3. **Lattice properties of the ToNV polyhedra**. (A/B) The two polyhedrin molecules are shown with the residues which mediate the interface shown. Regions outside of the interface have been removed or made transparent for clarity. Residues which interact with the adjacent protomer are shown as sticks and grouped according to whether they participate in electrostatic or hydrophobic interactions across the interface. (C) Integrity of the native or two different recombinant polyhedrin structures. Coloured from the N-terminus (blue) to C-terminus (red). (D) Comparison of lattices between the native and expanded recombinant lattices.

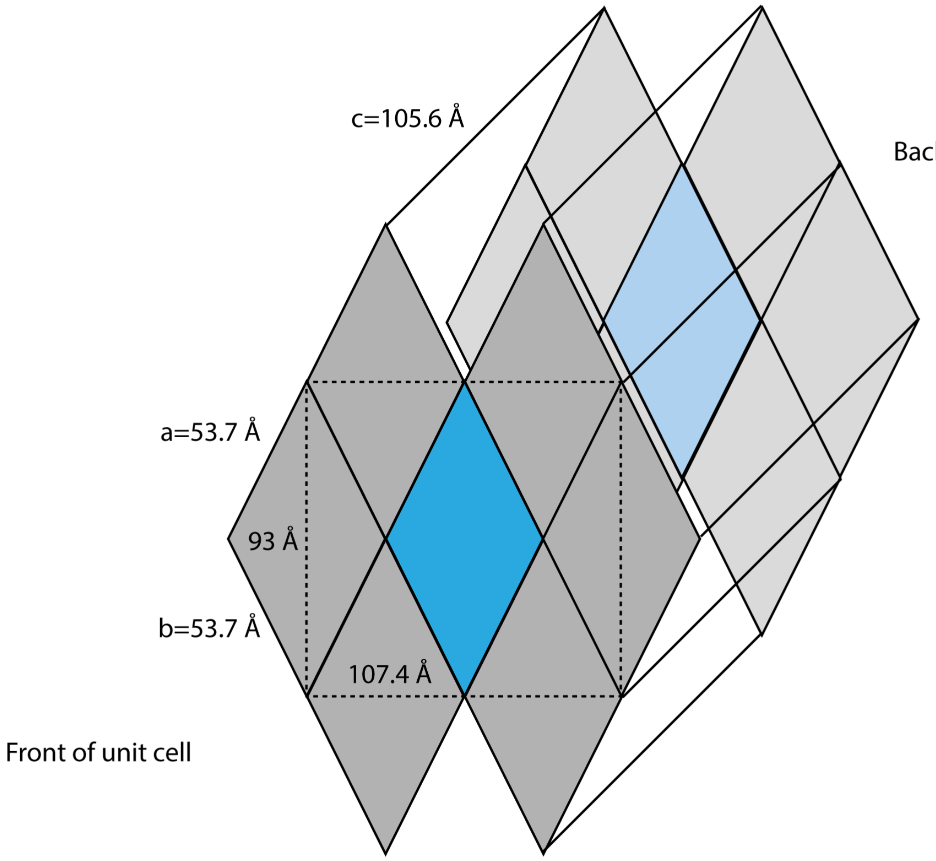

Supplementary Figure 4. **Alternative index of the lattice.** The $P32_12$ unit cell (shown in shades of blue) has unit cell dimensions of a = b = 53.7 Å and c = 105.6 Å. The front and back of neighbouring unit cells are shown in shades of grey. By indexing the crystal differently, we can generate an orthorhombic lattice that has close to equal unit cell dimensions of a = 93 Å, b = 107.4, and c = 105.6.

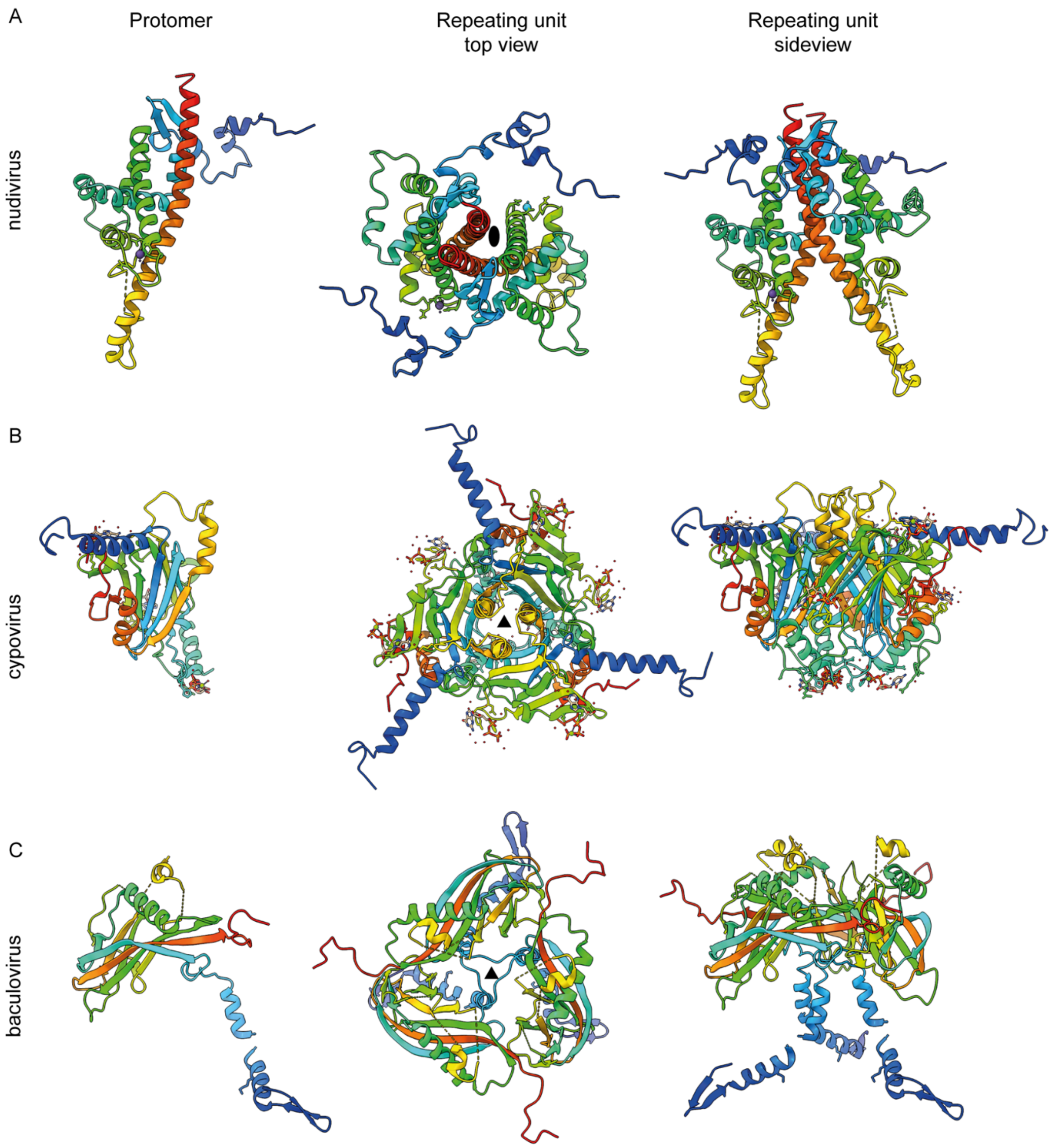

Supplementary Figure 5. **Examples of viral polyhedrin.** Polyhedrin structure of the protomer or repeating unit for (A) *Nudiviridae*, (B) *Baculoviridae* (PDB ID: 3JVB from *Wiseana signata* nucleopolyhedrovirus), and (C) cypovirus (PDB ID: 2OH5 from *Bombyx mori* cypovirus *1*). The models are coloured from N-terminus (blue) to C-terminus (red). Nucleotides and waters are shown as sticks and spheres, respectively.

	Native ToNV OB	Recombinant ToNV OB	Recombinant ToNV OB-Expanded cell	SeMet Recombinant ToNV OB
Beamline	DLS VMXm	DLS I24	DLS I24	DLS VMXm
Number of crystals	16	289	10	55
Wavelength (Å)	0.6328	1.5498	1.5498	0.9787
Resolution range (Å)	28.0-1.69 (1.75-1.69)	46.57-1.96 (2.03-1.96)	44.87-2.8 (2.9-2.8)	34.77-1.91 (1.98-1.91)
Space group	$P3_221$	$P3_221$	$P3_221$	$P3_221$
Unit cell (a,b,c) (Å)	53.4, 53.4, 105.6	53.8, 53.8, 105.8	57.2, 57.2, 105.8	53.5, 53.5, 105.3
Unique reflections	19056 (1962)	13279 (1297)	4961 (478)	13277 (1129)
Multiplicity	19.6 (15.9)	160.7 (21.5)	3.0 (2.9)	51.7 (2.9)
Completeness (%)	94.0 (99.6)	100 (99.6)	93.8 (93.7)	93.9 (79.7)
$\langle I/\sigma(I)\rangle$	9.9 (1.9)	16.0 (0.9)	6.4 (2.3)	24.6 (2.6)
Wilson B-factor	16.1	28.1	45.1	17.7
R_{pim}	0.18 (2.38)	0.040 (2.309)	0.15 (0.49)	0.035 (0.74)
$CC_{1/2}$	0.97 (0.32)	0.99 (0.53)	0.96 (0.28)	0.95 (0.28)
CC*	0.99 (0.70)	1 (0.83)	0.99 (0.66)	0.99 (0.66)
Anomalous slope				2.125
R_{work}	0.18 (0.29)	0.18 (0.28)	0.25 (0.28)	0.17 (0.24)
R_{free}	0.21 (0.30)	0.23 (0.36)	0.27 (0.30)	0.22 (0.35)
CC_{work}	0.96 (0.79)	0.95 (0.81)	0.90 (0.74)	0.95 (0.68)
CC_{free}	0.95 (0.77)	0.94 (0.77)	0.88 (0.69)	0.89 (0.69)
No. of atoms				
Macromolecules	1839	1722	1260	1707
Ligands (Ca)	1	1	-	1
Solvent	125	70	12	101
Protein residues	230	215	161	213
R.m.s.d., bond lengths (Å)	0.005	0.004	0.006	0.005
R.m.s.d., bond angles (°)	0.66	0.64	0.77	0.77
Ramachandran				
Favoured (%)	97.8	97.2	95.4	98.1
Allowed (%)	2.2	2.8	4.6	1.9
Outliers (%)	0	0	0	0
Average B-factor				
Overall	18.4	36.1	38.4	21.6
Macromolecules	18.0	36.0	38.5	21.2
Ligands (Ca)	14.7	30.3	-	16.9
Solvent	24.2	38.7	33.8	29.1
PDB ID	8BBT	8BCK	8BCL	8BC5

Supplementary table 1. **Data collection and refinement statistics. Values in parentheses are for the high resolution shell.**
