# Supplementary material for: Atomic structure of a nudivirus occlusion body protein determined from a 70-year-old crystal sample
Source: Nat Commun. 2023 Jul 13;14:4160. doi: 10.1038/s41467-023-39819-1 (PMC10345106; doi:10.1038/s41467-023-39819-1)
Supplement: Supplementary file 3 — Reporting Summary [file 41467_2023_39819_MOESM3_ESM.pdf]

nature portfolio

Corresponding author(s): Jeremy Keown and Jonathan Grimes

Last updated by author(s): Jun 20, 2023

Reporting Summary

Nature Portfolio wishes to improve the reproducibility of the work that we publish. This form provides structure for consistency and transparency in reporting. For further information on Nature Portfolio policies, see our Editorial Policies and the Editorial Policy Checklist.

Statistics

For all statistical analyses, confirm that the following items are present in the figure legend, table legend, main text, or Methods section.

n/a	Confirmed	
☒	☐	The exact sample size (n) for each experimental group/condition, given as a discrete number and unit of measurement
☒	☐	A statement on whether measurements were taken from distinct samples or whether the same sample was measured repeatedly
☒	☐	The statistical test(s) used AND whether they are one- or two-sided<br>*Only common tests should be described solely by name; describe more complex techniques in the Methods section.*
☒	☐	A description of all covariates tested
☒	☐	A description of any assumptions or corrections, such as tests of normality and adjustment for multiple comparisons
☐	☒	A full description of the statistical parameters including central tendency (e.g. means) or other basic estimates (e.g. regression coefficient) AND variation (e.g. standard deviation) or associated estimates of uncertainty (e.g. confidence intervals)
☒	☐	For null hypothesis testing, the test statistic (e.g. F, t, r) with confidence intervals, effect sizes, degrees of freedom and P value noted<br>*Give P values as exact values whenever suitable.*
☒	☐	For Bayesian analysis, information on the choice of priors and Markov chain Monte Carlo settings
☒	☐	For hierarchical and complex designs, identification of the appropriate level for tests and full reporting of outcomes
☒	☐	Estimates of effect sizes (e.g. Cohen's d, Pearson's r), indicating how they were calculated

Our web collection on statistics for biologists contains articles on many of the points above.

Software and code

Policy information about availability of computer code

Data collection	GDA
Data analysis	DIALS v1, xia2.multiplex v1, coot v0.98, Phenix v1.19-1.21, chimeraX v1.4-1.6, PyMOL 2.4.1, CRANK 1.5, trRosetta v1, AlphaFold 2 (as Collab fold) v1.5

For manuscripts utilizing custom algorithms or software that are central to the research but not yet described in published literature, software must be made available to editors and reviewers. We strongly encourage code deposition in a community repository (e.g. GitHub). See the Nature Portfolio guidelines for submitting code & software for further information.

Data

Policy information about availability of data

All manuscripts must include a data availability statement. This statement should provide the following information, where applicable:

- Accession codes, unique identifiers, or web links for publicly available datasets
- A description of any restrictions on data availability
- For clinical datasets or third party data, please ensure that the statement adheres to our policy

All data are available from the corresponding authors and/or included in the paper or Supplementary information. Crystallographic data generated in this study have been deposited in the PDB including accession codes 8BBT [http://doi.org/10.2210/pdb8BBT/pdb] (archival sample), 8BC5 [http://doi.org/10.2210/pdb8BC5/pdb] (selenomethionine data), 8BCK [http://doi.org/10.2210/pdb8BCK/pdb] (recombinant data), and 8BCL [http://doi.org/10.2210/pdb8BCL/pdb] (recombinant

data-expanded cell). Source data are provided with this paper. Data generated in previous studies (PDB ID: 3JVB [http://doi.org/10.2210/pdb3JVB/pdb] and PDB ID: 2OH5 [http://doi.org/10.2210/pdb2OH5/pdb] are available in the PDB.

Human research participants

Policy information about studies involving human research participants and Sex and Gender in Research.

Reporting on sex and gender	N/A
Population characteristics	N/A
Recruitment	N/A
Ethics oversight	N/A

Note that full information on the approval of the study protocol must also be provided in the manuscript.

Field-specific reporting

Please select the one below that is the best fit for your research. If you are not sure, read the appropriate sections before making your selection.

☒ Life sciences ☐ Behavioural & social sciences ☐ Ecological, evolutionary & environmental sciences

For a reference copy of the document with all sections, see nature.com/documents/nr-reporting-summary-flat.pdf

Life sciences study design

All studies must disclose on these points even when the disclosure is negative.

Sample size	For X-ray crystallography experiments, sample size was not pre-calculated. Instead data was collected until strong signal above the noise was achieved in the on-the-fly processing. This sample size was deemed sufficient as it gave enough signal to solve and determine the structures.<br><br>For SDS-PAGE experiments, duplicate sample were run and showed similar results.
Data exclusions	Data was excluded from crystals with weak diffraction.
Replication	For SDS-PAGE experiments, duplicate sample were run and showed similar results. Recombinant crystal production was carried out at least two times and diffraction was similar for both preparations.
Randomization	Randomization of samples is not appropriate for these experiments as we needed to know which samples were being collected. During processing of the X-ray crystallography data a random 5% of reflections were not used in refinement to instead be used in validation.
Blinding	Blinding was not appropriate as decisions had to be made on the correct type of analysis for the different samples.

Reporting for specific materials, systems and methods

We require information from authors about some types of materials, experimental systems and methods used in many studies. Here, indicate whether each material, system or method listed is relevant to your study. If you are not sure if a list item applies to your research, read the appropriate section before selecting a response.

Materials & experimental systems

n/a	Involved in the study	
☒	☐	Antibodies
☐	☒	Eukaryotic cell lines
☒	☐	Palaeontology and archaeology
☒	☐	Animals and other organisms
☒	☐	Clinical data
☒	☐	Dual use research of concern

Methods

n/a	Involved in the study	
☒	☐	ChIP-seq
☒	☐	Flow cytometry
☒	☐	MRI-based neuroimaging

Eukaryotic cell lines

Policy information about cell lines and Sex and Gender in Research

Cell line source(s)	SF9 cells were sourced from the Division of Structural Biology cell bank at the University of Oxford.
Authentication	Authentication was not performed for this study.
Mycoplasma contamination	Mycoplasma contamination was not performed for this study
Commonly misidentified lines (See ICLAC register)	No commonly misidentified cell lines were used in this study.
